# Computational Approaches for the Design of Novel Anticancer Compounds Based on Pyrazolo[3,4-d]pyrimidine Derivatives as TRAP1 Inhibitor

**DOI:** 10.3390/molecules26195932

**Published:** 2021-09-30

**Authors:** Amena Ali, Magda H. Abdellattif, Abuzer Ali, Ola AbuAli, Mohd Shahbaaz, Mohamed Jawed Ahsan, Mostafa A. Hussien

**Affiliations:** 1 Department of Pharmaceutical Chemistry, College of Pharmacy, Taif University, P.O. Box 11099, Taif 21944, Saudi Arabia; 2 Department of Chemistry, College of Science, Taif University, P.O. Box 11099, Taif 21944, Saudi Arabia; O.abuali@tu.edu.sa; 3 Department of Pharmacognosy, College of Pharmacy, Taif University, P.O. Box 11099, Taif 21944, Saudi Arabia; abuali@tu.edu.sa; 4 South African Medical Research Council Bioinformatics Institute, University of Western Cape, Private Bag X17, Bellville, Cape Town 7535, South Africa; mohammed@sanbi.ac.za; 5 Laboratory of Computational Modelling of Drugs, South Ural State University, 76 Lenin Prospects, 454080 Chelyabinsk, Russia; 6 Department of Pharmaceutical Chemistry, Maharishi Arvind College of Pharmacy, Ambabari Circle, Jaipur 302039, India; jawedpharma@gmail.com; 7 Department of Chemistry, Faculty of Science, King Abdulaziz University, P.O. Box 80203, Jeddah 21589, Saudi Arabia; maabdulaal@kau.edu.sa; 8 Department of Chemistry, Faculty of Science, Port Said University, Port Said 42521, Egypt

**Keywords:** TRAP1, 3D-QSAR pharmacophore modeling, TRAP1 kinase, virtual screening, molecular dynamics simulations

## Abstract

In the present in-silico study, various computational techniques were applied to determine potent compounds against TRAP1 kinase. The pharmacophore hypothesis DHHRR_1 consists of important features required for activity. The 3D QSAR study showed a statistically significant model with R^2^ = 0.96 and Q^2^ = 0.57. Leave one out (LOO) cross-validation (R^2^ CV = 0.58) was used to validate the QSAR model. The molecular docking study showed maximum XP docking scores (−11.265, −10.532, −10.422, −10.827, −10.753 kcal/mol) for potent pyrazole analogs (42, 46, 49, 56, 43), respectively, with significant interactions with amino acid residues (ASP 594, CYS 532, PHE 583, SER 536) against TRAP1 kinase receptors (PDB ID: 5Y3N). Furthermore, the docking results were validated using the 100 ns MD simulations performed for the selected five docked complexes. The selected inhibitors showed relatively higher binding affinities than the TRAP1 inhibitor molecules present in the literature. The ZINC database was used for a virtual screening study that screened ZINC05297837, ZINC05434822, and ZINC72286418, which showed similar binding interactions to those shown by potent ligands. Absorption, distribution, metabolism, and excretion (ADME) analysis showed noticeable results. The results of the study may be helpful for the further development of potent TRAP1 inhibitors

## 1. Introduction

TRAP1 (tumor necrosis factor (TNF) receptor-associated protein 1) is a 90 kDa protein that encodes the mitochondrial chaperone protein Heat Shock Protein (Hsp90) and is closely related to tumorigenesis promotion in a variety of cancers [1,2]. TRAP1 helps maintain mitochondrial integrity, thus smoothing the progression of cell death against cellular stresses, which is obtained by reduced ROS production and reprogramming cellular metabolism. These two factors (maintaining mitochondrial integrity and reduced ROS production) allow cancer cells to adapt better to harsh tumor microenvironments [3,4,5]. Furthermore, TRAP1 inactivation encourages cancer cells to undergo substantial apoptosis, in-vitro and in-vivo; hence, numerous targeting mitochondrial TRAP1 inhibitors have been developed [6].

**Figure 1:**
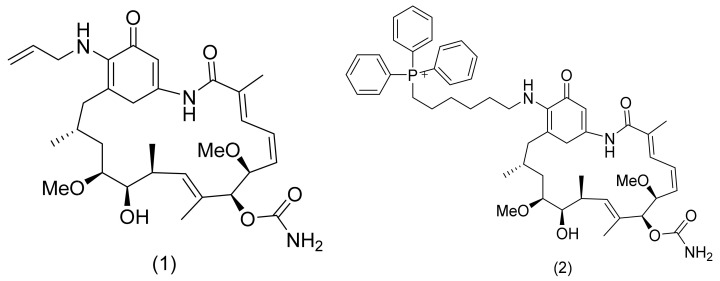
The 2D representation of **1** Tanespimycin and **2** Gamitrinibs.

There are several inhibitors of TRAP1, including tanespimycin (**1**) Figure 1 (17-AAG, CP127374, NSC-330507, KOS 953), a potential inhibitor with a maximal half inhibitory concentration (IC_50_) value of 5 nM, which has 100 times better activity in Hsp90-derived cells than in normal cells. However, it further induces necrosis, apoptosis, autophagy, and mitophagy. Gamitrinibs (**2**) is another drug that has shown potent activity against TRAP1 in prostate cancer patients [7,8].

TRAP1 is an imperative bioenergetic regulator because it can inhibit cytochrome oxidase and succinate dehydrogenase (SDH) [9]. TRAP1 also provides resistance to oxidative stress and counterbalances the permeability of mitochondrial transition and consequent cell death.

Various studies have shown the importance of TRAP1 in stress conditions, as it protects cells against ROS-induced apoptosis and senescence. Furthermore, TRAP1 mRNA and protein are highly expressed in cancer cell lines and tumors. Therefore, the present study focused on developing inhibitors targeting TRAP1 due to its role in cancer—computational studies designed these inhibitors before synthesizing compounds to minimize the time for new drug discovery.

In the present work, a computational study was performed for 34 different pyrazole analogs, as reported in the literature. Pharmacophore mapping was used to identify the important features of biological activity. The 3-D QSAR study by an atom-based model provided good statistical values with significant Q^2^ and R^2^ values. The generated pharmacophore features have been taken for virtual screening from the ZINC database.

Virtual screening studies provided information about the potential effects of various ZINC compounds against TRAP1, comparable to the dataset. Finally, molecular docking studies investigated the important molecular interactions with the TRAP1 active site for the different surrounding amino acids in combination with Molecular Dynamics (MD) simulations. This study provided the information to support the development of potent inhibitors.

## 2. Materials and Methods

### 2.1. Data Collection

**Figure 2:**
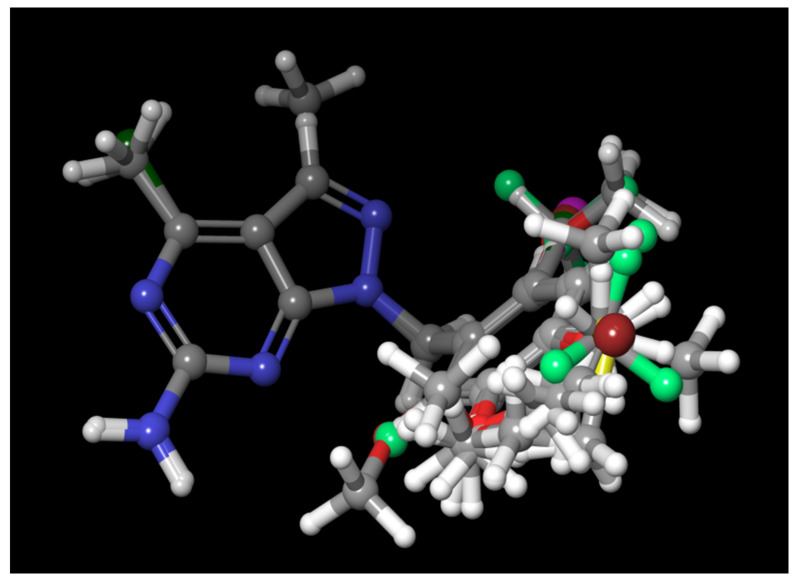
Alignment of common pharmacophoric features.

A dataset of 34 different pyrazolo[3,4-d]pyrimidine analogs was used to study the experimentation of TRAP1 inhibitory activities for synthesized molecules [10]. Chem-Draw Professional 16.0 software was used to sketch all the dataset structures saved in “.mol” format. The maximal half inhibitory concentration (IC_50_) in µM was converted to pIC_50_ (negative log of the IC_50_) for QSAR analysis. The common core of pyrazole analogs (1) and their different substituted groups are shown in Table 1. For the 3D-QSAR analysis, the entire dataset was divided into two sets (i.e., training and test sets) in 7:3 ratios to predict the pIC_50_ values. In addition, 5 partial least-squares (PLS) factor testing was carried out to derive the phase hypothesis. Ligand development was performed with the LigPrep module using Maestro v12.1, which helped generate input structures for pharmacophore Alignment and Scoring Engine (PHASE) and Grid-Based Ligand Docking from Energetics (Glide) modules. Different optimizations of output structures provided the various requirements of the simulation programs. Clean-up wizards can efficiently convert 2D structures into 3D structures and process one ligand per second, which helped in the docking study and pharmacophore development by using unique algorithms [11,12,13]. Different molecules processed through energy minimization and aligned on a common scaffold are described in Figure 2.

### 2.2. Study of Pharmacophore Development

The pharmacophore mapping study examined the common unique chemical features based on the structural specifications necessary to determine the biological activity. Schrödinger Maestro v12.1 PHASE module software was used for the pharmacophore mapping study [14]. Among the various hypotheses, DHHRR_1 was selected as it had the best pharmacophore characteristics.

In the current study, three common chemical features of the module were used, which included a hydrophobic group (H), ring aromaticity (R), and a hydrogen bond donor (D). A distance calculation tool was used to find the positions of these mentioned features, which are an innate part of the module providing the inter-feature distance map (Figure 2). The nitrogen atom of the pyrimidine ring acts as a donor atom, while the two Rs (as a benzene ring) are attached directly to the pyrazole ring. In addition, the nitrogen group displayed two hydrophobic interactions of H [15,16,17].

### 2.3. Pharmacophore Hypothesis Generation

**Figure 3:**
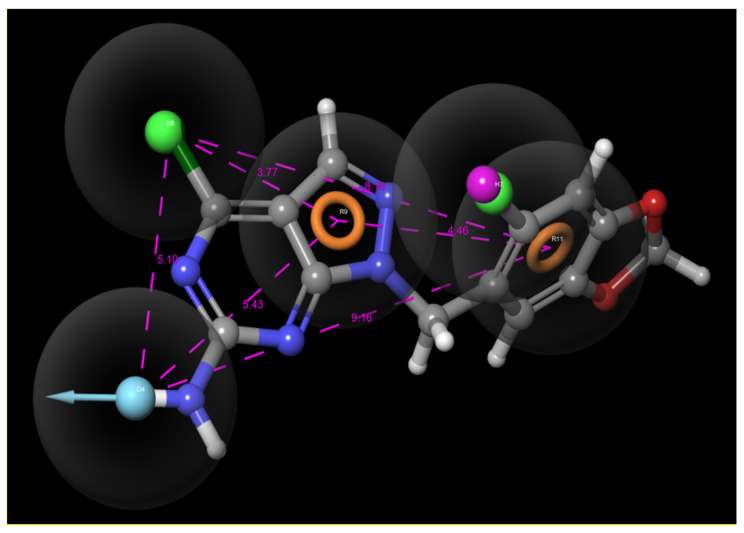
The best common pharmacophoric hypothesis.

The relation between the chemical features and structural similarities of the 34 mentioned compounds provided the opportunity to generate 20 possible hypotheses that can explain the binding ability of active molecules with receptors, having a box size of 1 Å and a 2 Å minimum inter-site distance. Thus, up to five features were set, which helped generate the maximum variants supporting establishing a common pharmacophore hypothesis [18]. The different parameters for the pharmacophore hypotheses include the following:

(1) Phase hypothesis score: rank-orders a new scoring function hypothesis, which helps provide knowledge of performance in virtual screening and the quality of ligand alignment and provides easy recognition of multiple binding modes by training against diverse known activities through the perception of a common pharmacophore; (2) Site score: helps provide the intimacy of superimposition of site points to the pharmacophore of the structure; (3) Survival score acts as blending terms for the number of matches, providing the relation between the relative energy and activity of the reference ligand; (4) Selectivity score: provides the negative logarithm of part of the molecules in the index and helps with matching the hypothesis; (5) Average outranking: actively adjusted rank minus one—outranking decoys are calculated for every docked active and averaged; (6) Receiver operating characteristic (ROC): aids in data analysis as an indicator of model performance, providing the differentiation of active sites from inactive compounds; (7) Vector score: average cosine of the angles between the analogous pairs of vector features (donors, acceptors, and aromatic rings) in different associated structures; (8) Active matched: provides knowledge of the number of active ligands matching the given hypothesis [19,20,21,22]. The best common pharmacophoric hypothesis is illustrated in Figure 3.

### 2.4. Model Development by Three-Dimensional QSAR Study

#### 2.4.1. Atom-Based QSAR

The atom-based QSAR method describes the molecule as spheres where different van der Waals radii overlap. The generated 3D-QSAR model is validated by the prediction of the activity by the test set ligands. The 3D-QSAR models were developed in Schrödinger Maestro v12.1 from a set of aligned ligands. Firstly, the energy minimized compounds with their activity have been imported into the phasing tool. The imported compounds have been divided into training and test sets based on a random selection where 70% of compounds were taken in the training set and 30% in the test set. Here we set criteria like grid spacing 1 Å, PLS factor of five, and the number of ligands to leave out 1. The elimination of variables has been performed by using a value less than two [23,24]. Then the models were evaluated in the value of R^2^, R^2^ CV, F, P, Q^2^, and so forth. Table 2 summarizes the different parameters of the QSAR model, which are as follows: Factors: number of various factors for partial least-squares regression model; SD: standard regression deviation; R^2^: value for regression; R^2^ CV: cross-validated R^2^ value calculated through the predictions acquired by a leave-N-out approach; F: variance ratio (larger F values represent higher statistically significant regressions); P: significance level of variance ratio (smaller values represent a greater degree of confidence); RMSE: root-mean-square error of the test set; Q^2^: for the predicted activities of the test set; Pearson’s r: for the predicted activities of the test set. The atom-type fraction segment displays the fraction due to each atom type in the QSAR model for each number of PLS factors used in the model. Confirmation of the least diversity in the biological activities between molecules of the training set through a scatter plot was obtained by plotting the actual activity against the predicted activity [25,26].

#### 2.4.2. Generation of Contour Maps

The contour maps helped predict the favorable or unfavorable interactions of aligned molecules with the receptor for biological activity and corresponded to the spatial arrangement of aligned molecules. For example, in the field-based model, regions with favorable steric fields are represented by green contours, and yellow contours represent unfavorable ones.

Moreover, the blue and red contours highlight positions where electropositive and electronegative groups would be positively connected, respectively. Thus, it is clear that biological activity will be more significant when there is more steric bulk near the green, less steric bulk near yellow, more positive charge near blue, and more negative charge near red. For the hydrogen bond donor contour map, a donor bulk near purple is favorable, but a donor bulk near cyan is unfavorable for more excellent biological activity. An acceptor bulk near red is desired for the hydrogen bond acceptor contour map, and an acceptor bulk near magenta is undesirable for improved biological activity [27]. In the atom-based model, blue cubes represent an increase in activity, and red-colored cubes represent a decrease in activity by a particular group. The contour maps can be described as follows:

The atom-based 3D-QSAR model visual representation: (a) electron-withdrawing; (b) hydrogen bond donor; (c) hydrophobic; and (d) positive ionic, where the positive coefficient (increase in activity) is represented as blue-colored cubes, while the negative coefficient (decrease inactivity) is represented as red-colored cubes.

Field contour maps: (a) electrostatic fields: blue as favored electropositive and red as disfavored electronegative; (b) hydrogen bond acceptor field: red as favored and magenta as disfavored; (c) hydrogen bond donor field: purple as favored and cyan as disfavored; (d) steric field: green as favored and yellow as unfavored.

#### 2.4.3. D-QSAR Model Evaluation

The 3D-QSAR model evaluation was carried out by considering key statistical parameters such as the squared cross-validation coefficient (Q^2^), the squared non-cross-validation coefficient (R^2^), predictive R^2^, and the standard error of estimate (SEE). The developed model was tested for internal quality based on the Q^2^ value, with an acceptance criterion of >0.5 being statistically significant for the model. The R^2^ provides the relative measure of the fit using a regression equation, with a value near 1.0 illustrating the best regression fit. Finally, the standard error of estimate conveys information about the variation of residuals or the regression line [28,29].

### 2.5. Virtual Screening Studies in the ZINC Database

The virtual screening study using the zinc database (https://zinc.docking.org/) was performed using pharmacophore hypothesis DHHRR_1 and the most active Compound **48** (Supplementary Materials Table S1). In the present study, we used the whole ZINC database in which only 7543 molecules were screened after applying the Lipinski rule of five. The ZINC database was used with drugs-like filters to download 7543 molecules. These molecules were screened by applying filters such as molecular weight, rotatable bond, RMSD values. The screened compounds of the ZINC database were further screened by virtual screening workflow. The workflow included filters like the Lipinski rule of five, which further screened the compounds before docking analysis. The final 4500 screened compounds were taken for HTVS docking analysis. Of the compounds from the top docking scores (kcal/mol), 50% were taken for SP docking analysis. The top 20% of compounds from SP were taken for XP docking analysis. The final top four hits are presented in the paper.

Target prediction was further performed using Swiss Target Prediction, a freely accessible tool for receptor databases (Supplementary Materials Table S2), through which the target exploration becomes more convenient and useful. Swiss Target Prediction was used to predict various protein targets, among which TRAP1 was the topmost suitable target for the different molecules considered. Thus, the screening of molecules was further performed through molecular docking with standard and extra precision modes against TRAP1 using the Glide module of Schrödinger [30].

### 2.6. Docking Study

Molecular docking studies on pyrazole analogs with TRAP1were carried out using Glide module software (Schrödinger Maestro v12.1). The Protein Data Bank (PDB ID:5Y3N) [31] was used for determining the protein structure, which was further processed through “protein preparation wizard’’ (Maestro wizard v12.1). Both the generating states and the refinement step were helpful in the automatic addition of atoms along with some important bonds at missing sites of protein molecules. The refinement step is crucial, as it is involved in optimizing H-bonded groups, dehydration, and restrained minimization by using default force field OPLS_ 3e. The processing of the receptor grid followed the completion of the optimization process to calculate the binding pocket of the receptors. The receptor grid had X, Y, and Z coordinates with 13.32, 56.37, and 0.13. These coordinates indicate the enclosing box where ligands molecules bind.

Various docked ligand conformations were observed in the docking results, showing their binding energy scores. Ranking based on scores provides a high rank for lesser scoring conformation [32].

### 2.7. Molecular Dynamics Simulations

The docked conformations with the highest binding affinities were subjected to MD simulations using GROMACS (version—2018-2) package [33]. The topology of the TRAP1 protein was generated using the GROMOS96 53a6 force field [34,35], and the parameterization of docked inhibitors was performed using the PRODRG server [36]. The correction of the partial charges of docked inhibitors was performed based on DFT theories present in the GAUSSIAN software suite, which uses the B3LYP 6-31G (d, p) basis set and CHELPG program [37]. After the topology generation, the system was solvated using the SPC/E water model [38] and then neutralized by adding an appropriate number of counter NA and CL ions. After this step, the energy minimization was performed using combined steepest descent, and conjugate gradient algorithms, with a convergence criterion, was set to around 0.005 kcal/mol. Afterward, the minimized systems were equilibrated by combining NVT (constant volume) and NPT (constant pressure) ensemble conditions, each for a period of 100 ps. The temperature of 300 K was maintained for the system using the Berendsen weak coupling method, and the pressure of 1 bar was maintained utilizing the Parrinello–Rahman barostat in the equilibration stage. The LINCS algorithm was used in the final production stages to generate structural conformational for a 100 ns timescale. The outputs were generated in the form of trajectories, which were analyzed to understand the behavior of each complex in the explicit water environment. The changes in the TRAP1-inhibitor distance, Hydrogen Bonds (H-bonds), Root Mean Square Deviations (RMSD), and Radius of Gyration (Rg) of the complex systems were analyzed. Furthermore, the Molecular mechanics Poisson–Boltzmann surface area (MM-PBSA) protocols implemented in the g_mmpbsa package [39] were used to calculate the attributes associated with binding free energy between TRAP1 and respective inhibitor molecules.

### 2.8. Absorption, Distribution, Metabolism, and Excretion (ADME) Property Predictions

ADME properties were determined using Swiss ADME and Schrödinger ADME online tools, which helped select ligands with drug-like properties. Lipinski (Pfizer) filter: implemented as MW ≤ 500, MLOGP ≤ 4.15, N or O ≤ 10, NH or OH ≤ 5 [40,41]. Ghose filter: implemented as 160 ≤ MW ≤ 480, −0.4 ≤ WLOGP ≤ 5.6, 40 ≤ MR ≤ 130, 20 ≤ atoms ≤ 70 [39,42]. Lead likeness: implemented as 250 ≤ MW ≤ 350, XLOGP ≤ 3.5, number of rotatable bonds≤ 7; Synthetic accessibility: from 1 (very easy) to 10 (very difficult) [43]. Default settings were employed for these calculations.

### 2.9. Enumeration Study

The R group enumeration module of Schrödinger was implemented for R-group-based enumeration of the pyrazolo scaffold. Drug-Like filters such as REOS and PAIN’s series were used for separating compounds with reactive functional groups. The obtained drug-like compounds were further processed for ligand preparation and the minimum energy with the help of the OPLS3e force field. Additionally, the docking of the final screened compounds was performed in the TRAP1 crystal structure in the ligand-binding cavity through the Glide SP protocol, resulting in docking poses. From these different docking poses, the 50 best poses were selected from other enumerations for further XP docking protocol analysis, providing the XP descriptors; this helped to describe the contributions of each atom in terms of penalties and rewards docking energy.

Enrichment calculations were performed for 1000 decoy compounds (from the DUD.E database) and 30 compounds (XP best poses) with the help of Schrödinger software, while docking was performed using the XP protocol. The obtained results helped to predict the validation of the docking protocol, with a Receiver Operating Characteristic (ROC) curve of R^2^ = 0.92.

## 3. Results and Discussion

### 3.1. Selection of Best Pharmacophore Hypothesis

All the selected compounds (compounds **1**–**34**) from the database were screened to obtain five probable standard pharmacophore features from the list of variants (i.e., two aromatic rings, two hydrophobic interactions, and one hydrogen bond donor). The mentioned features were presumed to have an essential role in the inhibitory ability of different compounds towards the target. Among the 20 hypotheses generated by the PHASE module, the DHHRR_1 hypothesis was considered the best by way of a scoring function mentioned in Table 2.

### 3.2. Pharmacophore Model Evaluation

**Figure 4:**
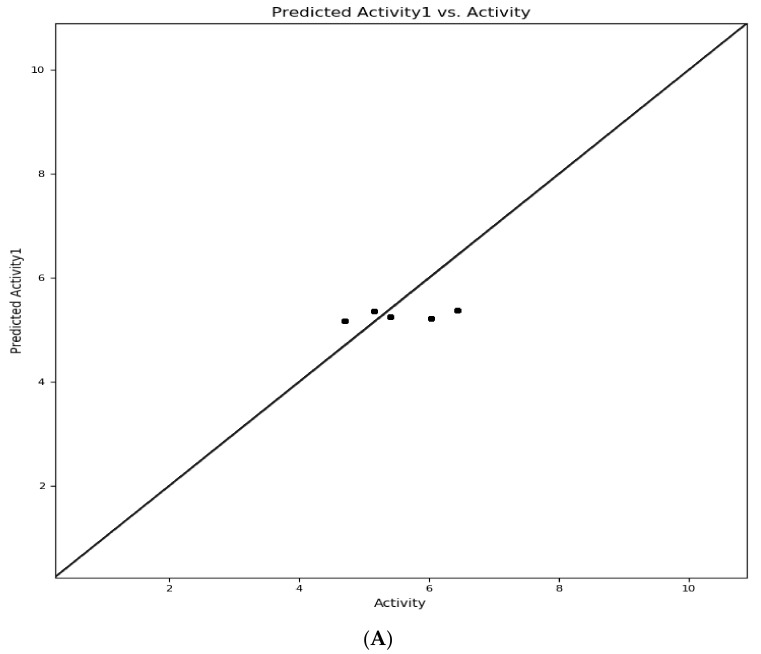

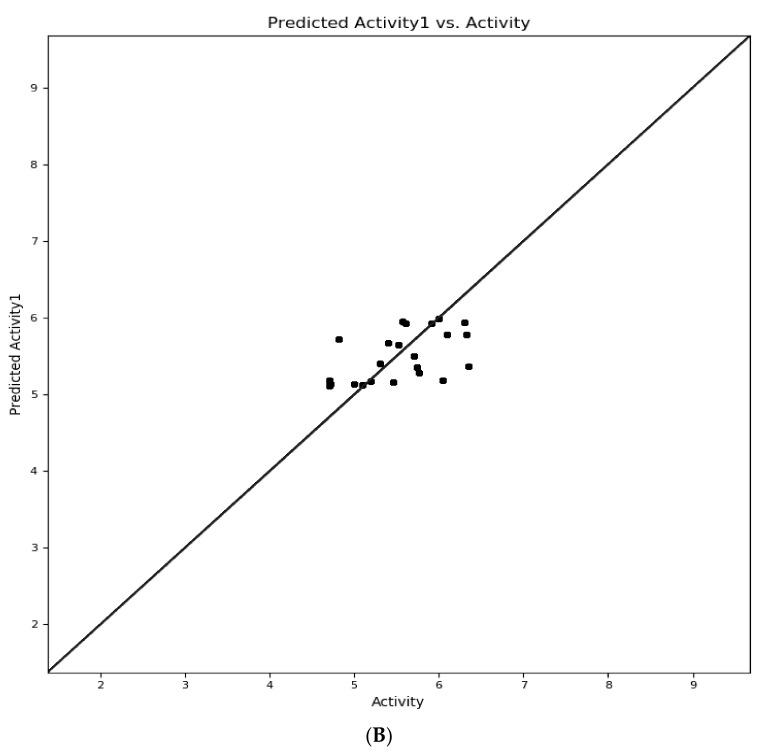
(**A**) Percent screen plot; (**B**) ROC plot.

The pharmacophore model’s quality was calculated using two evaluation tools: the percent screen plot and the ROC plot. Percent screen plot represents the percentage of actives recovered and the percentage of ligands screened for the hypothesis. The ROC plot is between the true-positive rate (sensitivity) and the specificity (specificity) for various cutoff points. A test is considered to have perfect discrimination when there is no overlap in two distributions. The test has an ROC curve representing 100% specificity and 100% sensitivity passing through the left upper corner. The closer the position of the curves in the left upper corner, the higher the overall accuracy of the method. Both the percent screen plot and the ROC plot were found to be in the extreme left corner, suggesting the better accuracy of the generated hypotheses by the PHASE module, as shown in Figure 4A,B.

### 3.3. Selection of Atom-Based QSARmodel

The QSAR results show the important statistics of the fit for both test and training sets. In Table 3, each row shows the hypothesis results. Lines within each row show regression models that have a specific value for least-squares factors, and PLS performed compound clustering with a factor of five. Different statistical parameters (SD, R^2^, P, F, Q^2^, RMSE, and Pearson’s *R*) in the QSAR model were considered for reliable predictions and evaluation of the QSAR model. The value of R^2^ is required, and a high R^2^ is essential for a model, but it alone does not provide the sufficient condition for ideal QSAR model prediction. Thus, predictive ability Q^2^ values have to be chosen to obtain the best QSAR model prediction. Based on these parameters, five different models were developed by modules and are shown in Table 3. Among the five models, the fifth model was significant due to higher values of 0.57, 0.96, and 0.58 for Q2, R2, and R2 CV values. Though higher values for SD (0.46) and RMSE (0.64) were recorded, shallow values of 0.08, 0.34, and 0.08 for Q^2^, R^2^, and R^2^ CV, respectively, diminished the probability of the first model. The required statistics for the atom-type fraction are reported in Table 4.

**Figure 5:**
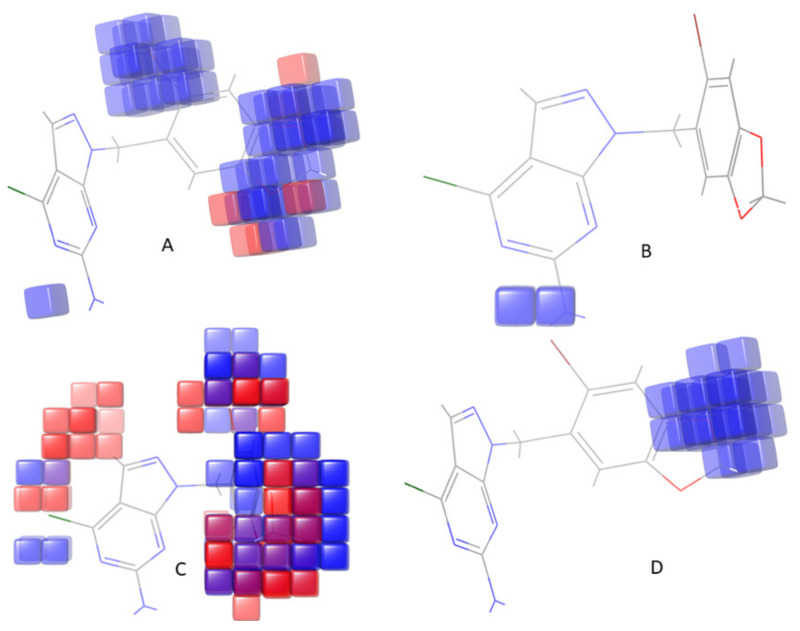
Atom-based 3D-QSAR model visual representation: (**A**) electron-withdrawing, (**B**) hydrogen bond donor, (**C**) hydrophobic, (**D**) positive ionic, where blue-colored cubes represent positive coefficients or an increase in activity, and red-colored cubes represent negative coefficients or decrease in the activity.

**Figure 6:**
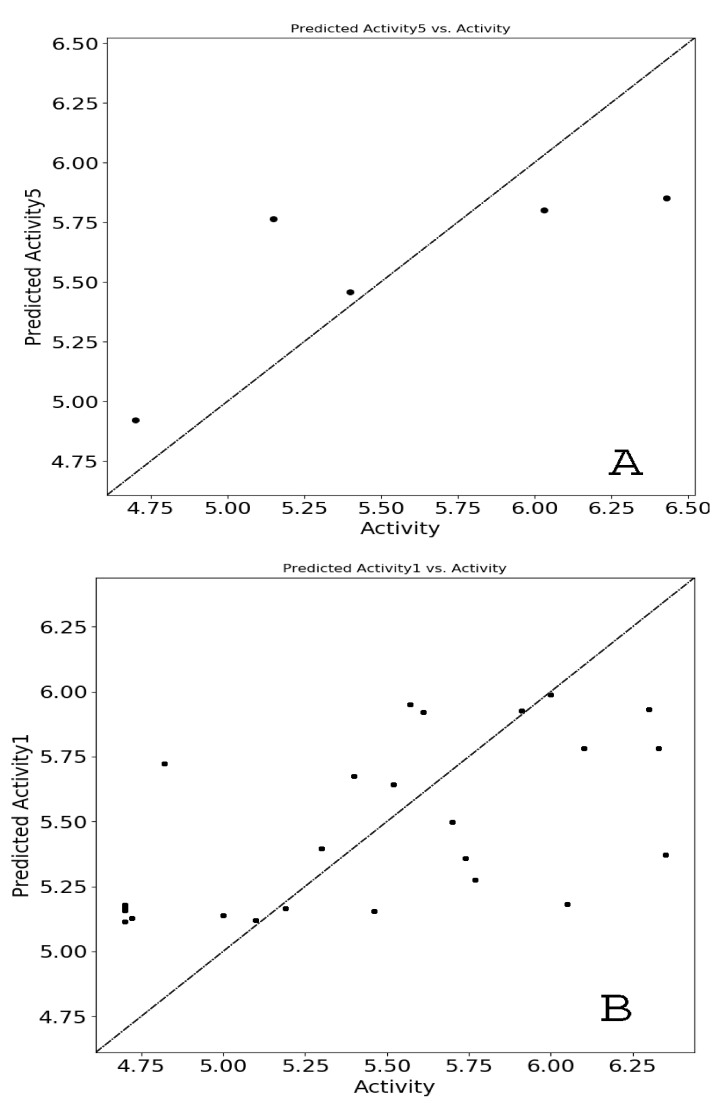
Comparison between actual vs. predicted pIC_50_ values of (**A**). test and (**B**). a training set molecules, consecutively.

Similarly, Table 5 presents the predicted pIC_50_, actual pIC_50_, and the residual models’ residual values. In addition, the atom-type fraction map provides information on the fractions of each atom of the training set affecting the activity and is shown in Figure 5. Finally, the uniform distribution of the training set obtained using a scatter plot of the displayed module passing through the origin (0, 0) as a straight line is shown in Figure 6. Again, R^2^ with an increased value with minimum Root Mean Square Error showed improved results with the removal of outliers.

### 3.4. Contour Map Analysis

Contour maps help to predict the biological activity and its correlation with various substituents on the core moiety (Figure 5) and help determine the effect of adding substituents to biological activity. An increase in biological activity is represented by a blue color, while a red color represents a decrease in biological activity in the occlusion map. Among the 34 compounds, the most active compound was selected based on the high survival value of DHHRR_1 of atom-based 3D-QSAR contour maps. An increase in activity is due to substitution of an electron-withdrawing group on the phenyl ring attached to pyrazolo[3,4-d]pyrimidine, suggesting that substitution of various groups such as -CN, -NO_2_, CF_3_, -NR_3_, -COR–X, and so forth, on the phenyl ring leads to augmented activity. Further, enhanced anticancer activity could be obtained by adding a hydrogen bond donor group at the pyrazolo[3,4-d]pyrimidine ring. Moreover, the hydrophobic group covers up the more significant part of the ring and is accountable for mixed activity.

### 3.5. Results of Molecular Docking

**Figure 7:**
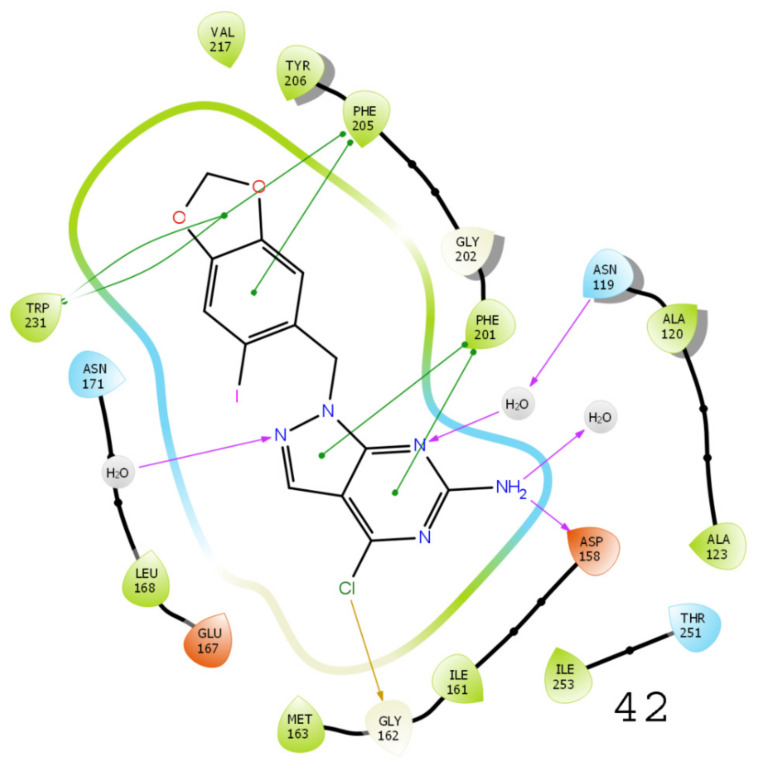

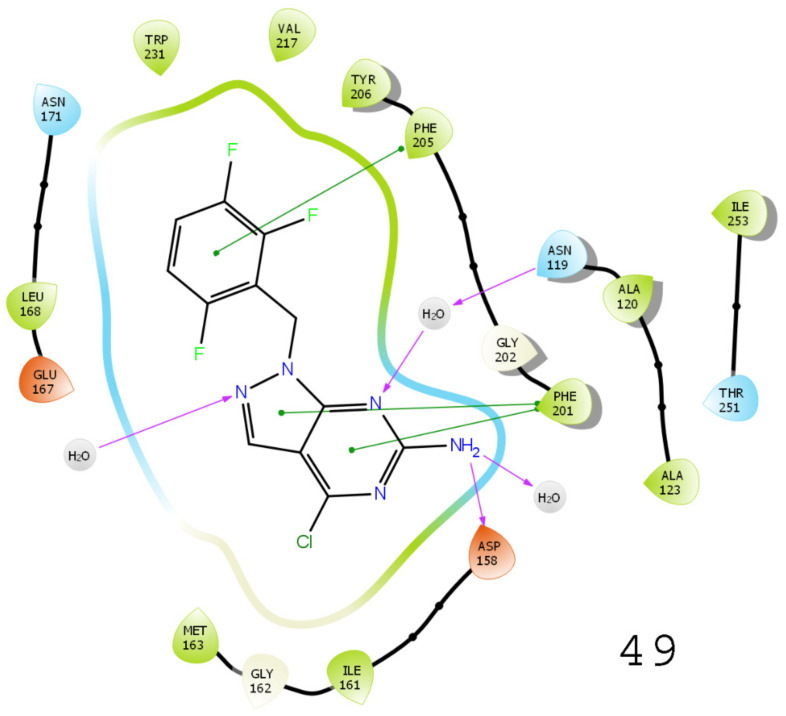
A 3D and a 2D diagram show binding interactions of compounds **42** and **49** with TRAP1 (PDB ID: 5Y3N).

**Figure 8:**
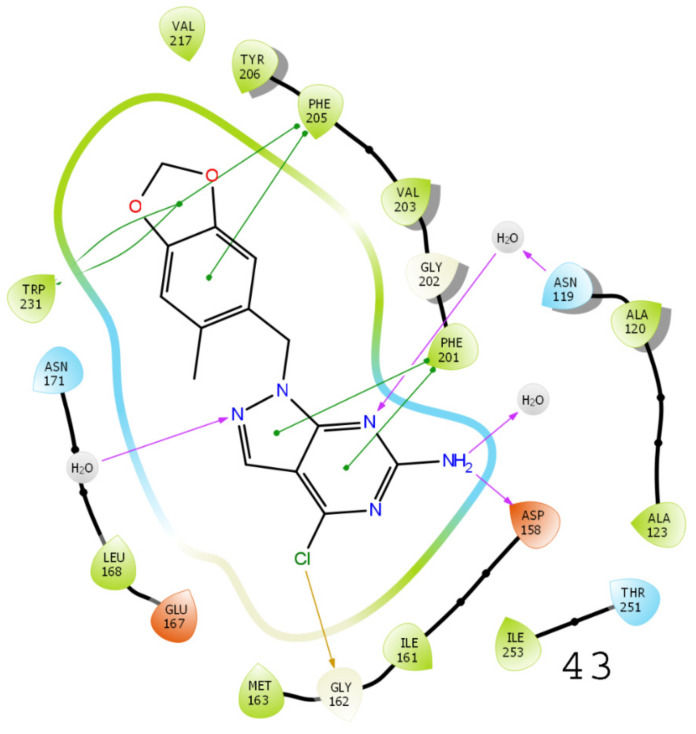

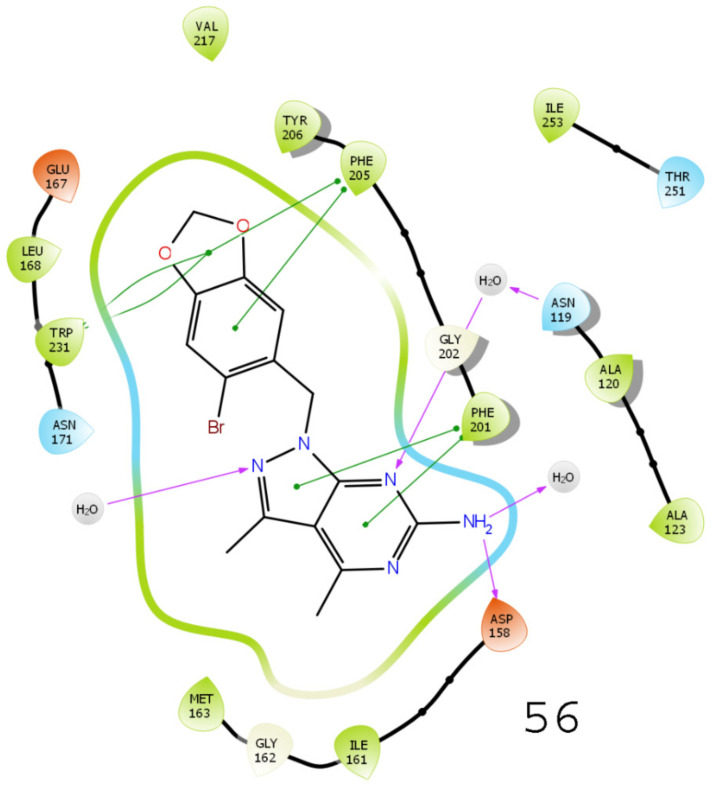
A 3D diagram showing binding interactions of compounds **43** and **56** with TRAP1 (PDB ID: 5Y3N).

A molecular docking study was performed to examine the possible interactions between protein and ligand molecules using the Schrödinger Glide module. The inhibition of enzyme activity depends on the possible interactions of inhibitors with various amino acid residues of the targeted protein of interest. Therefore, docking was performed for all compound’s analogs to study the binding cavity of TRAP1 (PDB ID: 5Y3N), the results of which are shown in Figure 7 and Figure 8. Purple-colored arrows and Π indicate the H-bonds–Π stacking interactions are characterized by purple-green-colored arrows. The possible bond interactions of compound 42 with amino acid residues PHE 201, GLY162, ASN119, ASP158, PHE205, and TRP231 were observed in this study. Similarly, the derivative compound 49 (XP docking scores value of −11.353 kcal/mol) was found to have possible critical interactions with PHE201, ASN119, ASP158, and PHE205 (Supplementary file S3). Further, the binding interactions of compound 43 were observed with PHE201, ASP158, GLY162, and PHE205, while in compound 56, interactions with PHE201, GLY202, and ASP158 amino acids were detected. These interactions are essential for TRAP1 inhibitory activity.

### 3.6. Results of Virtual Screening

**Figure 9:**
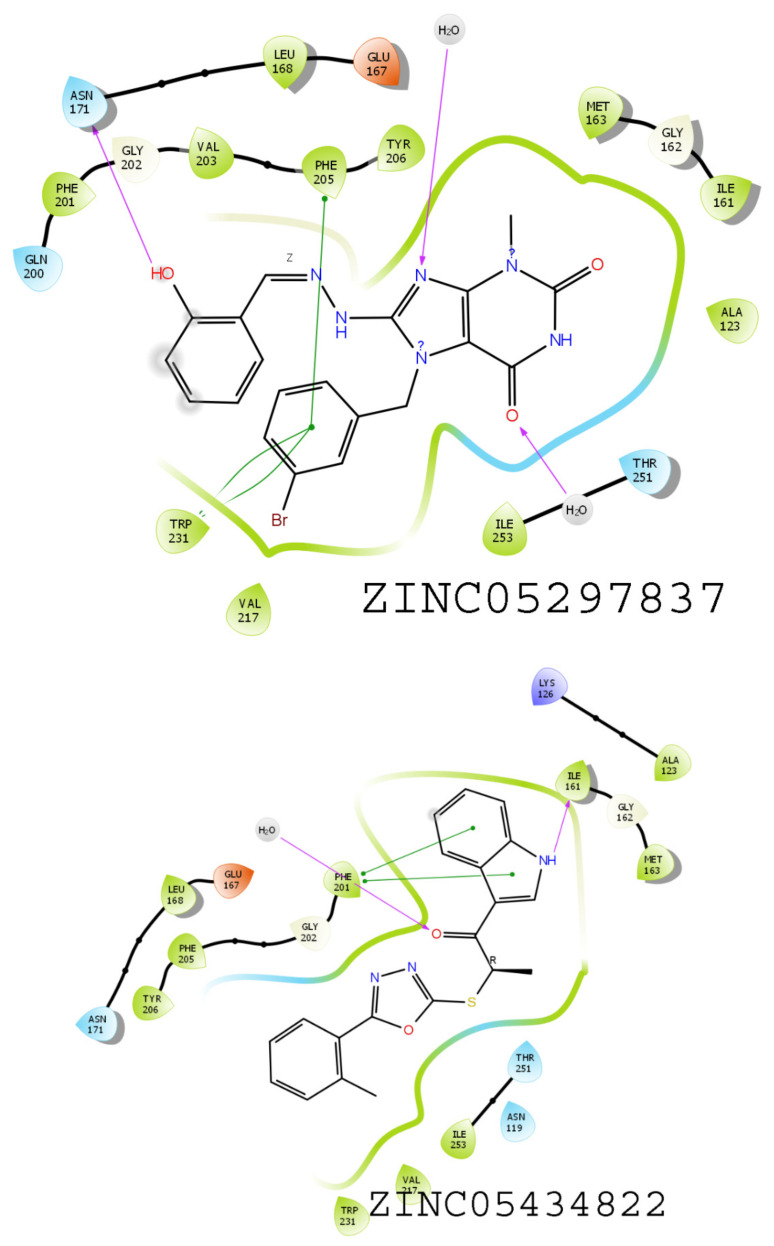
TRAP1(PDB ID:5Y3N) with ZINC05297837 and ZINC05434822 compounds showing binding interactions with amino acids.

**Figure 10:**
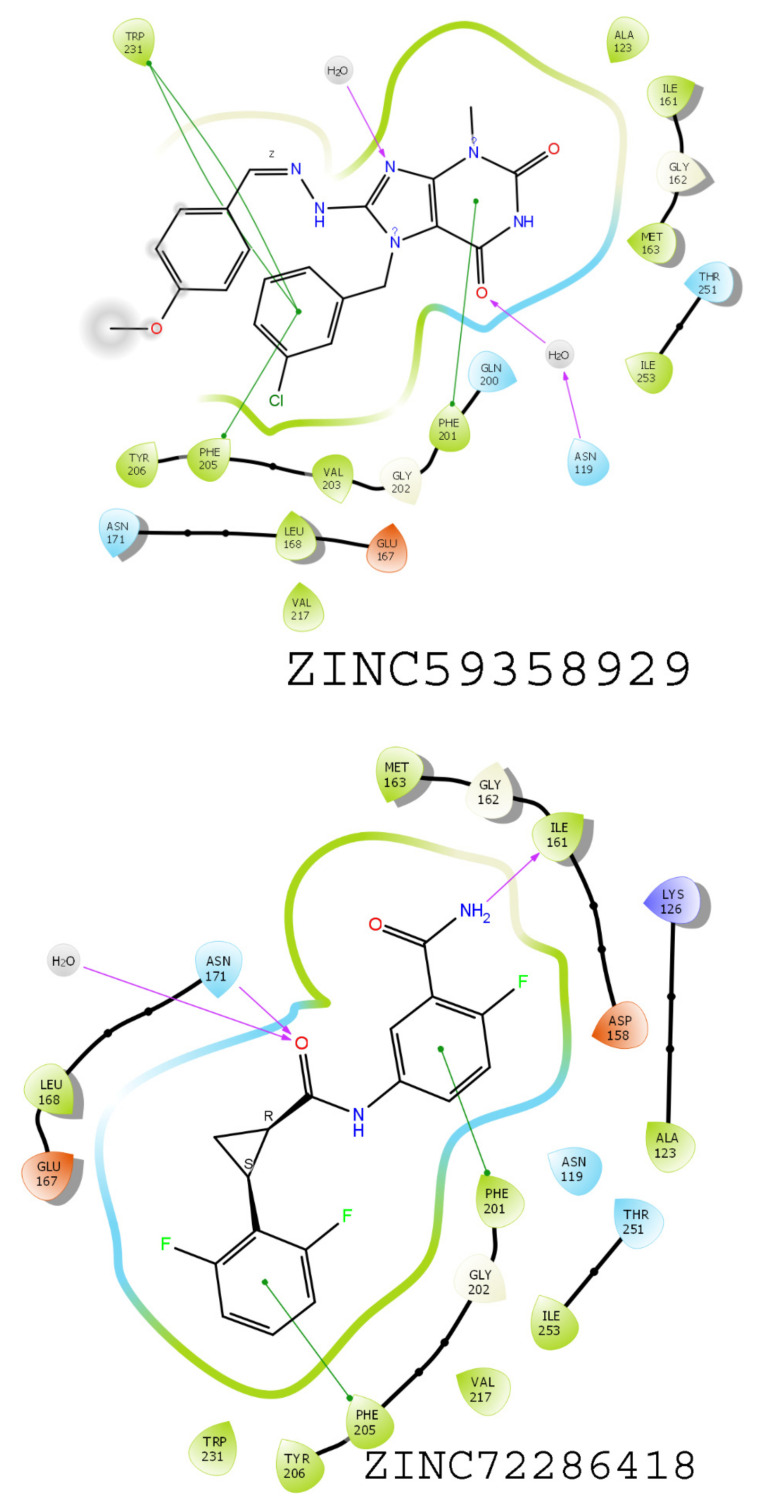
TRAP1(PDB ID:5Y3N) with compounds ZINC59358929 and ZINC72286418, showing binding interactions with amino acids.

The virtual screening study was performed utilizing pharmacophore hypothesis DHHRR_1 utilizing the ZINC database, resulting in the screening of 2832 compounds with the help of Lipinski’s rule of five. These screened compounds were further used in the high-throughput virtual screening (HTVS) docking methodology. The best 20% of the compounds from HTVS were subjected to SP docking. Similarly, the top 20% of the screened compounds from SP docking were further subjected to XP docking (Supplementary file S4). In total, 16 compounds were screened through SP docking, in which the top hits, namely, ZINC05434822, ZINC72286418, ZINC05297837, and ZINC59358929, were found to have docking scores of −11.97, −10.73, −9.98, and −9.88 kcal/mol, respectively. These compounds were taken into consideration for further study as the final ZINC compounds. These compounds were evaluated in terms of binding interaction energy by MMGBSA. Among these four compounds, ZINC05297837 showed interaction with amino acid residues PHE205, TRP231, and ASN171 via the phenyl ring (Figure 9). In contrast, ZINC05434822 showed interaction with PHE201 and ILE161 in the same cavity as the crystal ligand shows (Figure 9). The compound ZINC59358929 showed binding interactions with ASN119, PHE201, PHE205, and TRP231, which is considered significant for showing activity, while the compound ZINC72286418 showed substantial interactions with ILE161, PHE201, and PHE205 (Figure 10).

The binding pocket residues were similar to those obtained from the binding of active compounds 42, 46, 48, 49, and 56 and the crystal ligand. The docking simulation study was further validated by checking the RMSD value, less than 2 Å.

### 3.7. MMGBSA-Based Rescoring

The MMGBSA-based rescoring method was used for the calculation of binding free energy for ligands and ZINC hit compounds ZINC05434822, ZINC72286418, ZINC05297837, and ZINC59358929 (complex with PDB ID:5Y3N), which provide very high binding free energy, as ΔG bind = −58.2, −42.07, −59.752, and −48.2 kcal/mol, respectively (Table 6, Supplementary Materials Table S5).

### 3.8. Analyses of the Conformations Obtained through MD Simulations

**Figure 11:**
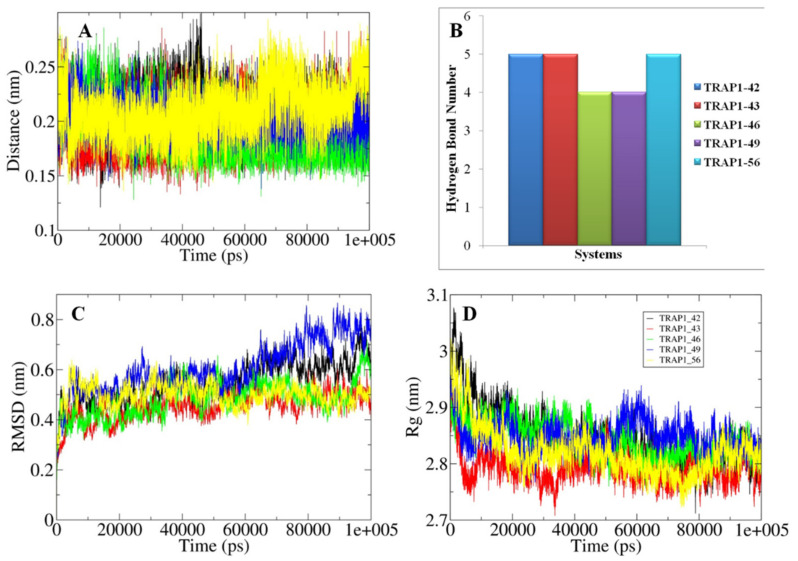
The graphical representation of MD simulation-based generated parameters with (**A**) shows the calculated distance changes between the docked TRAP1 and inhibitors. (**B**) Illustrating the variation observed in the hydrogen bonding during the 100 ns time scale. (**C**) Highlighting the changes in the RMSD values observed during MD simulations. (**D**) Projecting the variations in the structural compactness of the docked systems in terms of the radius of gyrations. (Black: TRAP1-42, Red: TRAP1-43, Green: TRAP1-46, Blue: TRAP1-49, Yellow: TRAP1-56).

**Figure 12:**
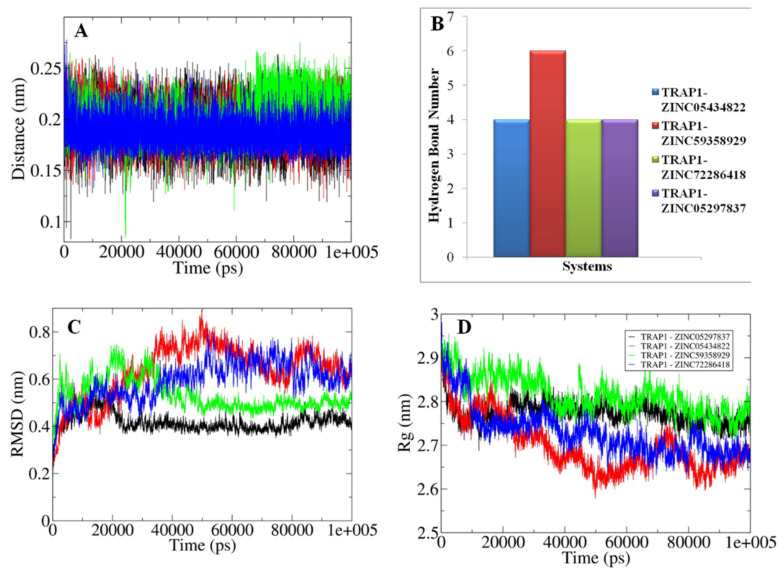
The diagrammatic representation of parameters generated after 100 ns MD simulations with (**A**) Illustrating the changes observed in the calculated distance between the docked TRAP1 and inhibitors. (**B**) Showing the variation observed in the hydrogen bonding patterns. (**C**) Projecting the changes in the RMSD values for different studies docked systems. (**D**) Highlighting the variations in the extent of a radius of gyrations. (Black: TRAP1-ZINC05297837, Red: TRAP1-ZINC05434822, Green: TRAP1-ZINC59358929, Blue: TRAP1-ZINC72286418).

The validation of the outcomes generated from the molecular docking and the changes in their structural attributes associated with each docked system were explored using the principles of MD simulations for the time scale of 100 ns. The nature of the bonding between the TRAP1 and the studied inhibitors was primarily assessed in terms of the calculated distances and hydrogen bonds (Figure 11A,B and Figure 12A,B). All the docked inhibitors showed a relatively similar closeness as observed from the calculated distances fluctuating between 0.15–0.25 nm. Furthermore, the docked complexes TRAP1-ZINC05434822 showed up to six H-bonds while in TRAP1-42, TRAP1-43, and TRAP1-49 systems, docked molecules form at least five H-bonds as compared to the rest of the complexes, which contain around four H-bonds. These observations indicate that the former systems showed the presence of a relatively higher bonding pattern.

Furthermore, the structural compactness and stability of the docked systems were analyzed in terms of the calculated RMSD and Rg values (Figure 11C,D and Figure 12C,D). The RMSD values highlighted the presence of relatively higher structural stability in the TRAP1-43 and TRAP1-ZINC05297837 systems in which equilibration was obtained somewhat earlier, and values were observed around 0.4 nm. Similarly, a slightly higher degree of compactness was observed in the TRAP1-43 and TRAP1-ZINC05434822 systems, inferred from the calculated Rg values fluctuating around 2.8 nm compared to the rest of the studied systems. These observations showed in the TRAP1-43 and TRAP1-ZINC05297837 and TRAP1-ZINC05434822 systems that the inhibitor binding to the protein leads to higher structural stability.

Moreover, the MMPBSA based calculations showed that relatively comparable binding affinity was observed in TRAP1-43 and TRAP1-46 systems as well as in TRAP1_ZINC05297837 and TRAP1_ZINC05434822 as indicated from the calculated binding energy than the rest of the studied systems (Table 7). The listed parameters were compared with the known inhibitor” NVP-AUY922”, whose information was collected from the literature [41]. These observations showed that, in the TRAP1_ZINC59358929 system, the inhibitor bounded to the studied protein with relatively higher affinity than the other systems.

### 3.9. Prediction of ADME Properties

The ADME properties were determined using Schrödinger ADME and Swiss ADME tools to obtain the best scoring of the dataset and ZINC compounds, as shown in Table 8 and Table 9. All compounds showed significant ADME properties, such as number of hydrogen bond donors (0–3), number of hydrogen bond acceptors (7), number of rotatable bonds (4–9), the molecular weight of <500, and molar refractivity of about 125, which are considerable (Supplementary files S6 and S7). The lipophilicity profile of the selected compounds represents the lipophilic character and high GI absorption. Still, none of the studied compounds were found to possess the ability to cross the blood–brain barrier, representing the lack of toxicity of the selected compounds. Compound ZINC72286418 was found to be soluble, as determined by the solubility profile of the ZINC-derived compounds, while others were moderately soluble in water. The synthetic convenience of all the compounds was in a good range (Supplementary files S5–S8).

## 4. Optimization of Novel Ligands

**Figure 13:**
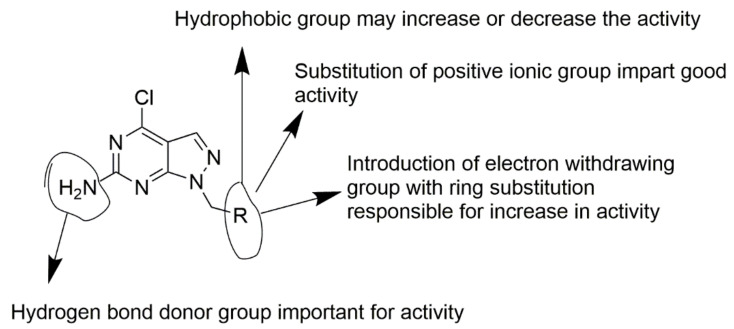
Ligand core with key features obtained by 3D-QSAR study for the development of novel molecules.

The optimization and development of novel TRAP1 inhibitors can be performed by using 3D-QSAR and molecular docking studies. Here, the results obtained by the 3D-QSAR analysis have been graphically represented as the structure–activity relationships (SARs) of the pyrazolo[3,4-d]pyrimidine core with different possible substituents (Figure 13).

## 5. Analysis of R Group Enumeration

Based on the optimized structure, several derivatives were enumerated through an R group enumeration study using Schrödinger software. The compound structures are described in Table 10, along with their XP docking scores. These compounds are novel derivatives of pyrazolo[3,4-d]pyrimidine, which has good docking scores.

## 6. Conclusions

In the present study, pharmacophore hypothesis development, QSAR, virtual screening and an enumeration study were performed to determine potential inhibitors against TRAP1. The best hypothesis generated was DHHRR_1, which was used for a virtual screening study employing the ZINC database. After many trials, the 3D QSAR study determined the best statistical values by changing the training and test set molecules. The resultant contour maps determined electrostatic, hydrogen bond acceptor, hydrogen bond donor, and positive ionic participation inactivity. The docking study of potent pyrazole analogues (42, 46, 49, 56, 43) showed the highest XP docking scores (−11.265, −10.532, −10.422, −10.827, −10.753 kcal/mol). The docking study showed that interaction with amino acids, such as PHE 583, CYS 532, SER 536, ASP 594, is important for activity.

Moreover, the MD simulations provided detailed structural insight and validated that the listed compounds were inhibiting the TRAP1 protein with relatively higher affinity. The ADME properties showed the important physicochemical properties of the molecules. The virtual screening study performed using the zinc database produced compounds ZINC05434822, ZINC72286418, and ZINC05297837, which showed essential binding interactions with receptor TRAP1 (PDB ID: 5Y3N). Correlating the docking results with the 3DQSAR analysis can provide more potential compounds as TRAP1 inhibitors. The enumeration of different positions of pyrazole analogs producing compounds with the best docking scores may be used for synthesis in research laboratories.

## Figures and Tables

**Table 1:** Different substituents of a common core with biological activities in IC_50_ and pIC_50_ values [10].

R_1_	Compounds	R	IC_50_ (µM)	pIC_50_
1	**4**	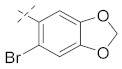	0.50	6.30
2	**9**	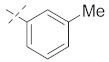	19.00	4.72
3	**10**	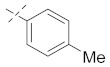	7.00	5.15
4	**11**	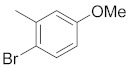	15.00	4.82
5	**12**	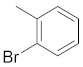	20.00	4.70
6	**13**	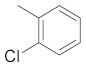	20.00	4.70
7	**15**	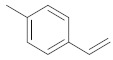	20.00	4.70
8	**22**	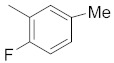	6.50	5.19
9	**23**	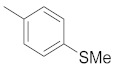	5.00	5.30
10	**24**	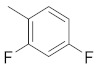	20.00	4.70
11	**25**	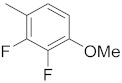	4.00	5.40
12	**26**	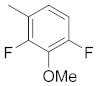	20.00	4.70
13	**27**	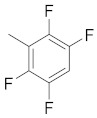	3.50	5.46
14	**30**	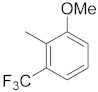	20.00	4.70
15	**32**	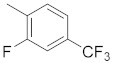	15.00	4.82
16	**33**	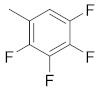	20.00	4.70
17	**34**	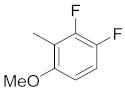	4.00	5.40
18	**35**	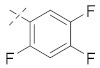	10.00	5.00
19	**36**	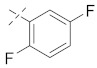	8.00	5.10
20	**39**	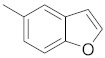	3.00	5.52
21	**41**	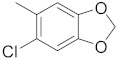	1.23	5.91
22	**42**	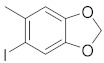	0.44	6.36
23	**43**	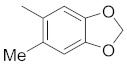	2.43	5.61
24	**44**	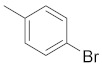	1.80	5.74
25	**45**	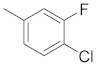	1.69	5.77
26	**46**	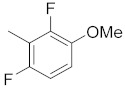	0.47	6.33
27	**47**	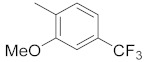	1.98	5.70
28	**48**	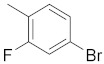	0.37	6.43
29	**49**	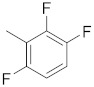	0.89	6.05
30	**50**	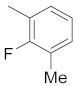	0.94	6.03
31	**51**	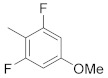	0.79	6.10
32	**52**	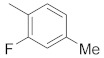	0.45	6.35
33	**56**	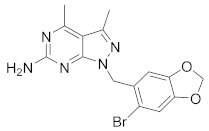	2.70	5.57
34	**59**	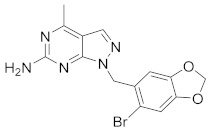	1.00	6.00

**Table 2:** Different pharmacophore hypotheses generated by using the compounds and their activity.

Hypo ID	Survival	Site	Vector	Volume	Select	Matches	Inactive	Adjusted	BEDROC	Ref. Lig
DHHRR_1	5.94	0.93	1.00	0.91	2.15	9.00	2.66	3.28	1.00	mol_32
DHHRR_2	5.94	0.93	1.00	0.91	2.15	9.00	2.62	3.33	1.00	mol_32
DHHRR_3	5.94	0.93	1.00	0.91	2.15	9.00	2.68	3.26	1.00	mol_32
DHHRR_4	5.94	0.92	1.00	0.91	2.15	9.00	2.70	3.24	1.00	mol_32
DHHRR_5	5.94	0.93	1.00	0.91	2.15	9.00	2.63	3.30	1.00	mol_32
DHHRR_6	5.94	0.93	1.00	0.91	2.15	9.00	2.70	3.24	1.00	mol_32
DHHRR_7	5.93	0.92	1.00	0.91	2.15	9.00	2.75	3.19	1.00	mol_32
DHHRR_8	5.93	0.92	1.00	0.91	2.15	9.00	2.65	3.29	1.00	mol_32
DHHRR_9	5.93	0.93	1.00	0.91	2.14	9.00	2.64	3.29	1.00	mol_32
DHHRR_10	5.93	0.93	1.00	0.91	2.14	9.00	2.67	3.26	1.00	mol_32
DHRR_1	5.40	0.99	1.00	0.94	1.52	9.00	2.90	2.50	1.00	mol_26
DHRR_2	5.40	0.99	1.00	0.94	1.52	9.00	2.90	2.50	1.00	mol_26
DHRR_3	5.39	0.99	1.00	0.94	1.51	9.00	2.89	2.50	1.00	mol_26
DHRR_4	5.39	0.99	1.00	0.94	1.51	9.00	2.91	2.48	1.00	mol_26
DHRR_5	5.39	0.99	1.00	0.94	1.51	9.00	2.90	2.49	1.00	mol_26
DHRR_6	5.39	0.99	1.00	0.94	1.51	9.00	2.89	2.50	1.00	mol_26
DHRR_7	5.39	0.99	1.00	0.94	1.51	9.00	2.91	2.48	1.00	mol_26
DHRR_8	5.39	0.99	1.00	0.94	1.50	9.00	2.90	2.48	1.00	mol_26
DHRR_9	5.39	0.99	1.00	0.94	1.50	9.00	2.90	2.48	1.00	mol_31
DHRR_10	5.38	0.99	1.00	0.94	1.50	9.00	2.91	2.47	1.00	mol_31

**Table 3:** Statistical data of atom-based QSAR model.

# Factors	SD	R^2^	R^2^ CV	R^2^ Scramble	Stability	F	RMSE	Q^2^	Pearson’s *r*
1.00	0.46	0.34	0.08	0.31	0.93	11.80	0.64	0.08	0.47
2.00	0.33	0.68	0.22	0.51	0.73	23.90	0.52	0.29	0.59
3.00	0.23	0.85	0.41	0.69	0.68	38.70	0.47	0.42	0.67
4.00	0.16	0.93	0.57	0.76	0.71	70.50	0.41	0.56	0.76
5.00	0.13	0.96	0.58	0.81	0.70	82.50	0.40	0.57	0.79

**Table 4:** 3D-QSAR statistics for the atom-type fraction.

# Factors	H-Bond Donor	Hydrophobic/Nonpolar	Electron-Withdrawing	Other
1	0.011	0.577	0.391	0.021
2	0.007	0.741	0.225	0.027
3	0.035	0.703	0.216	0.046
4	0.043	0.738	0.198	0.021
5	0.045	0.755	0.192	0.008

**Table 5:** Predicted pIC_50_ and residual values of generated models.

No ^r^	Name	Actual pIC_50_ (X)	Atom-Based	
			Predicted pIC_50_ (Ϋ)	Residuals (Ϋ−X)
1	4	6.3	6.00	−0.30
2	9	4.72	4.65	−0.07
3	10	5.15	5.76	0.61
4	11	4.82	4.85	0.03
5	12	4.7	4.66	−0.04
6	13	4.7	4.69	−0.01
7	15	4.7	4.68	−0.02
8	22	5.19	5.20	0.01
9	23	5.3	5.30	0.00
10	24	4.7	4.85	0.15
11	25	5.4	5.29	−0.11
12	26	4.7	4.67	−0.03
13	27	5.46	5.60	0.14
14	30	4.7	4.92	0.22
15	32	4.82	4.88	0.06
16	33	4.7	4.63	−0.07
17	34	5.4	5.46	0.06
18	35	5	4.89	−0.11
19	36	5.1	5.14	0.04
20	39	5.52	5.57	0.05
21	41	5.91	5.98	0.07
22	42	6.36	6.39	0.03
23	43	5.61	5.73	0.12
24	44	5.74	5.85	0.11
25	45	5.77	5.75	−0.02
26	46	6.33	6.11	−0.22
27	47	5.7	5.86	0.16
28	48	6.43	5.85	−0.58
29	49	6.05	6.02	−0.03
30	50	6.03	5.80	−0.23
31	51	6.1	6.11	0.01
32	52	6.35	6.20	−0.15
33	56	5.57	5.63	0.06
34	59	6	6.17	0.17

**Table 6:** Docking scores of active compounds and ZINC screened compounds with their MMGBSA scores.

Compound Name	PDB ID:5Y3N		
	Docking Score (Extra-Precision (XP)) kcal/mol	Docking Score (Standard Docking Precision (SP)) kcal/mol	MMGBSA ΔG Bind (XP Complex) kcal/mol
**48**	−10.824	−9.998	78.07
**42**	−11.265	−11.265	−57.88
**46**	−10.532	−10.782	−56.71
**49**	−10.422	−11.353	−68.2
**56**	−10.827	−11.641	−82.07
**43**	−10.753	−11.508	−56.71
ZINC05434822	−11.63	−11.641	−68.2
ZINC72286418	−10.86	−10.59	−82.07
ZINC05297837	−10.42	−6.25	−59.752
ZINC59358929	−10.102	−9.68	−78.2

**Table 7:** The list of attributes associated with the free energy of binding between the TRAP1 and studied inhibitors.

S. No	Docked Complex	MM-PBSA Based Calculated Energies (kJ/mol)			
		ΔE (vdW)	ΔE (Elec)	ΔG (SASA)	ΔG (Binding)
1	NVP-AUY922	−133.245	−20.399	−17.746	−276.797
2	TRAP1_42	−174.918	−6.766	−16.056	−213.795
3	TRAP1_43	−190.049	−22.551	−15.727	−244.055
4	TRAP1_46	−201.654	−12.756	−16.761	−247.931
5	TRAP1_49	−178.876	−16.962	−15.849	−227.536
6	TRAP1_56	−184.313	−16.979	−16.403	−234.098
7	TRAP1_ZINC05297837	−268.296	−2.926	−19.745	−310.711
8	TRAP1_ZINC05434822	−277.273	−13.936	−18.528	−328.265
9	TRAP1_ZINC59358929	−303.182	−43.700	−22.525	−391.933
10	TRAP1_ZINC72286418	−219.998	−12.829	−17.952	−268.731

**Table 8:** ADME predictions of ZINC database and other active compounds. Predicted 1: Octanol/water partition coefficient; 2: Caco-2 cell permeability (nm/s); 3: Brain/blood partition coefficient; 4: Apparent MDCK cell permeability (nm/s); 5: Human serum albumin binding]; 6: Number of metabolic reactions; 7: Percent human oral absorption.

S. No.	Compound Name	QP log Po/w	QPP Caco	QP logBB	QPPMDCK	# Metab	QP logKhsa	Percent Human Oral Absorption
1	**48**	3.156	920.499	−0.145	4124.662	1	0.106	100
2	**42**	2.644	1011.799	−0.193	2743.625	1	−0.039	96.212
3	**46**	2.972	1006.302	−0.295	2031.339	2	0.025	100
4	**49**	2.892	1037.573	−0.101	3427.589	1	0.027	100
5	**56**	2.686	1271.458	−0.292	1230.569	3	0.1	100
6	**43**	2.354	1053.419	−0.305	1260.235	2	−0.053	94.825
7	ZINC05434822	4.687	1015.224	−0.589	833.988	3	0.728	100
8	ZINC72286418	2.653	431.141	−0.802	581.552	3	0.1	89.635
9	ZINC05297837	2.728	258.436	−1.334	303.018	2	0.003	86.094

**Table 9:** Physicochemical property prediction of ZINC database and other active compounds.

S. No.	Name	Mol. Wt. (g/mol)	No. Rot. Bonds	No. H-Bond Acceptors	No. H-Bond Donors	Molar Refractivity
1	**48**	356.58	2	4	1	78.14
2	**42**	429.6	2	5	1	89.27
3	**46**	325.7	3	6	1	76.89
4	**49**	313.67	2	6	1	70.36
5	**56**	376.21	2	5	1	89.17
6	**43**	317.73	2	5	1	81.52
7	ZINC05434822	363.43	5	4	1	102.8
8	ZINC72286418	334.29	5	5	2	80.71
9	ZINC05297837	469.29	5	5	3	118.83

**Table 10:** R group determination by enumeration study using the Schrödinger software.

Comp. Name	Structure	XP GS Score (PDB ID:5Y3N)	R1 s m Smiles	R2 s m Smiles	R3 s m Smiles
**1**	O=C(C)Nc(n1)nc([NH^+^](C)C)c(c12)cnn2Cc(c(c3)C(=O)N)cc(c34)OCO4	−13.286	[*][NH+](C)C	[*]NC(=O)C	[*]C(=O)N
**2**	n1c([NH3^+^])nc(O)c(c12)cnn2Cc(c(c3)C(=O)N)cc(c34)OCO4	−13.286	[*]O	[*][NH3+]	[*]C(=O)N
**3**	C1C[NH2^+^]CCC1c(nc(n2)C(=O)N)c(c23)cnn3Cc(c(c4)C(=O)N)cc(c45)OCO5	−12.873	[*]C1CC[NH2+]CC1	[*]C(=O)N	[*]C(=O)N
**4**	NC(=O)c(n1)nc(O)c(c12)cnn2Cc(cc(c34)OCO4)c(c3)C(=O)Nc5ccccc5	−12.73	[*]O	[*]C(=O)N	[*]C(=O)Nc1ccccc1
**5**	NC(=O)c(n1)nc(O)c(c12)cnn2Cc(cc(c34)OCO4)c(c3)-c5[nH]ccn5	−12.674	[*]O	[*]C(=O)N	[*]c1ncc[nH]1
**6**	NC(=O)c(n1)nc(O)c(c12)cnn2Cc(cc(c34)OCO4)c(c3)-c5[nH]cnc5	−12.622	[*]O	[*]C(=O)N	[*]c1cnc[nH]1
**7**	CNC(=O)Nc(n1)nc(O)c(c12)cnn2Cc(cc(c34)OCO4)c(c3)NC(=O)Nc5ccccc5	−12.608	[*]O	[*]NC(=O)NC	[*]NC(=O)Nc1ccccc1
**8**	n1c([NH3+])nc(O)c(c12)cnn2Cc(c(c3)C(=O)N(C)C)cc(c34)OCO4	−2.955	from water 1	15173	15183
**9**	c1nccn1-c(n2)nc(O)c(c23)cnn3Cc(cc(c45)OCO5)c(c4)C(=O)Nc6ccccc6	−12.559	[*]O	[*]n1ccnc1	[*]C(=O)Nc1ccccc1
**10**	n1c([NH3^+^])nc([NH2^+^]C)c(c12)cnn2Cc(cc(c34)OCO4)c(c3)C(=O)Nc5ccccc5	−12.555	[*][NH2+]C	[*][NH3+]	[*]C(=O)Nc1ccccc1

## Data Availability

The data presented in this study are available on request from the corresponding author.

## Supplementary Materials

The glade files and docking files for the compounds are available in Supplementary Materials.

## Sample Availability

Samples of the compounds are not available from the authors.
